# Assessment of knowledge, attitudes and practices towards anthrax in Narok County, Southern Kenya

**DOI:** 10.11604/pamj.2021.38.120.19439

**Published:** 2021-02-03

**Authors:** Josephat Mutiso Mbai, Jack Owiti Omolo, Dominic Wamamba, Daniel Maritim, Zeinab Gura, Mark Obonyo

**Affiliations:** 1 Field Epidemiology and Laboratory Training Program, Ministry of Health, Nairobi, Kenya,; 2 Department of Health, County Government of Narok, Narok, Kenya

**Keywords:** Anthrax, transmission, attitude, outbreak, Kenya

## Abstract

**Introduction:**

anthrax is endemic in some parts of Kenya causing mortalities in livestock and morbidity in humans. On January 20^th^, 2018, news media reported suspected anthrax in a remote southern Kenyan village after villagers became ill following consumption of meat from a dead cow that was confirmed, by microscopy, to have died of anthrax. We assessed community knowledge, attitude and practices (KAP) to identify intervention gaps for anthrax prevention.

**Methods:**

we conducted a KAP survey in randomly selected households (HHs) in villages from selected wards. Using multi-stage sampling approach, we administered structured questionnaire to persons aged ≥15 years to collect KAP information from February 11^th^-21^st^, 2018. From a set of questions for KAP, we scored participants’ response as “1” for a correct response and “0” for an incorrect response. Univariate analysis and Chi-square tests were performed to explore determinants of KAP. Concurrently, we gathered qualitative data using interview guides for thematic areas on anthrax KAP from key informant interviews and focus group discussions. Qualitative data were transcribed in Ms Word and analyzed along themes by content analysis.

**Results:**

among 334 respondents: 187/334 (56%) were male; mean age, 40.7±13.6 years; 331/334 (99.1%) had heard of anthrax and 304/331 (91.8%) knew anthrax to be zoonotic. Transmission was considered to be through eating dead-carcasses by 273/331 (82.5%) and through contact with infected tissue by 213/331 (64.4%). About 59% (194/329) regularly vaccinated their livestock against anthrax, 53.0% (174/328) had slaughtered or skinned a dead-animal and 59.5% (195/328) practiced home slaughter while 52.9% (172/325) treated sick-animals by themselves. Sex (p≤0.001), age (p=0.007) and livestock-rearing years (p≤0.001) were significantly associated with knowledge and practice.

**Conclusion:**

there were differences in knowledge and practices towards anthrax by age-group and sex. Enhanced public health education and targeted interventions by relevant government agencies is recommended.

## Introduction

Anthrax is an infectious disease primarily of herbivores caused by a gram-positive, aerobic, spore-forming, and rod-shaped bacterium, *Bacillus anthracis* [1]. Anthrax occurs worldwide with a varying incidence in different geographical regions and is considered endemic in some parts of Africa [2]. In Kenya, anthrax is listed as one of the priority zoonoses of public health significance [3]. It causes human and livestock morbidity, mortality and leads to reduced trade in livestock and livestock-derived products [4]. Anthrax outbreaks are regular occurrences among livestock populations. Of Kenya´s 47 counties, 19 (40%) reported both human and animal anthrax cases between 2012 through 2017 [1,2]. Majority of human cases occur in agricultural environment as a result of individuals coming into contact with dead or dying animals [1].

On January 20, 2018, news media reported suspected anthrax in a remote village in Trans Mara East sub-county, Narok County, southern Kenya after several villagers became ill after consuming meat from a dead cow. Telephone inquiries to the sub-county public health and veterinary authorities confirmed the media reports. The local community described the disease as *“burasta”* loosely translated to mean *curse of sudden death in livestock*. Its outbreak meant the administrative authorities are notified at once! Veterinary records confirmed positive blood smears for anthrax for cattle carcasses that were destroyed following the outbreak. Past interventions in the affected area had included community education to prevent meat consumption of suspected animal anthrax cases. Kenya´s Ministry of Health (MOH) dispatched a team of medical, veterinary and laboratory epidemiologists to investigate this outbreak in February 2018. We aimed to assess community knowledge, attitudes and practices (KAP) towards anthrax to identify intervention gaps for anthrax prevention and control.

## Methods

**Figure 1:**
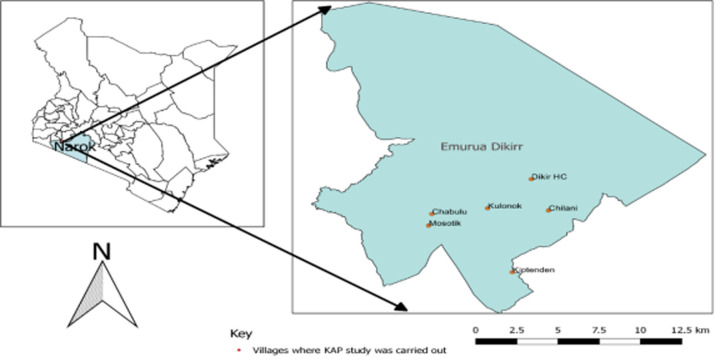
map of Kenya showing Trans Mara East sub-county investigation sites, 2018

**Investigation site**: Trans Mara East is one of the six sub-counties of Narok County. It is the most densely populated with an estimated 132,816 human population within an area of 320.50 square kilometers (Figure 1). The topography consists of acacia trees and shrubs in low lying areas. The soils are clay loam rich in magnesium and calcium plant nutrients. Illiteracy level in Narok County stands at 36%, one of the highest in Kenya [5]. The sub-county is divided into four wards namely: Ilkerin, Ololmasani, Mogondo and Kapsasian. It neighbors Maasai Mara national park and livestock production consists mainly of indigenous breeds reared in free grazing lands. Estimated livestock population consists of 80,800 heads of cattle, 62,000 sheep and goats, 6,500 donkeys and 120,000 poultry.

**Survey design and population**: utilizing a cross-sectional study design, we collected KAP information from February 11^th^, 2018 through February 21^st^, 2018 involving both quantitative and qualitative survey tools: questionnaire; focus group discussions (FGD) and key informant interviews (KII). To participate, an individual had to be aged ≥15 years and above, been a resident of the investigation area for at least one year and did or did not keep livestock. Individuals of unsound mind and those who declined were excluded from the survey.

### Quantitative survey

**Sample size determination and sampling method**: using a single proportion Cochran formula and assuming 88% proportion for anthrax awareness and design effect of two, we targeted 326 households (HHs) for inclusion in the survey [4,6]. We utilized multi-stage sampling strategy to enroll KAP respondents. To ascertain outbreak and non-outbreak areas, the team first visited the sub-county health and veterinary authorities and purposely selected two wards owing to logistics and time constraints. Of the two wards, a full list of villages and respective numbers of HHs in each village was obtained from the health authorities. A village was the smallest administrative unit of a county in Kenya. We allocated HHs to be sampled proportionate to the HH numbers in the two wards. Mosotik village where the outbreak had occurred and four other villages were randomly selected from the list. Thereafter, survey respondents were selected through simple random sampling and HHs for interview were subsequently selected from the first household by skipping five households. In every selected household, a consenting household head or in his/her absence the senior-most resident of the household who was over 15 years was selected for interview. If the selected household did not have anybody present during the visit or did not have a person meeting the inclusion criteria or declined to be interviewed, the next immediate household was visited. This process was done until the minimum sample size was achieved.

**Data collection**: using an interviewer administered structured questionnaire, we collected information on socio-demographics (age, sex, and education), information on knowledge (causes, transmission, clinical symptoms, prevention and control); attitude (opinion about anthrax disease) and practices (meat consumption, slaughter and skinning of dead animals and treatment seeking behaviors). The data collection tools were moderated after piloting in eight households around Dikirr village which shared similar characteristics to the investigation area.

**Data management**: survey data were entered, cleaned and analyzed using Microsoft^®^ Excel (Microsoft Office, Seattle, USA) and Epi Info^TM^ 7 (CDC, 1600 Clifton Rd, Atlanta, GA 30333, USA). We performed univariate analysis for socio-demographics and characteristics associated with knowledge, attitude and practices. Overall knowledge on anthrax was assessed through a set of 34 questions related to knowledge on anthrax (cause, species affected, transmission routes, signs and symptoms, and prevention and control). Similarly, 16 item questions were used to assess good practice (livestock vaccination; management of sick livestock; actions following sudden death; home slaughter; meat inspection services; and consumption of dead animal). We allotted participants´ response as “1” for a correct response and “0” for an incorrect response. To assess for knowledge and practices on anthrax, individual respondent scores were summed for variables pertaining to these items. The median was calculated, each for knowledge and practice and the median used as the cut off score. Respondents with a score less than the median for knowledge and practice were considered to have inadequate knowledge/poor practice. We performed Chi-square or Fishers-exact tests to explore association between socio-demographic characteristics, knowledge scores and practice scores. Statistical significance was set at p-value < 0.05.

**Qualitative data collection**: qualitative research tools were used; FGDs and in-depth interview with key informants. Each FGD targeted between 8-12 participants. The FGD participants were purposely invited through local administration, public health and veterinary authorities. Discussions were conducted using interview guides focused on thematic areas for anthrax knowledge, attitude and practices as well as challenges in anthrax control. Discussions were held in Kenya´s national languages and translated with the assistance of field assistants. Data was collected through note taking, audio-recording and photographs. In-depth interviews were held with veterinary officers, public health officers, clinicians and local administration. We sought their insight and interactions with the local communities while implementing anthrax control programs, achievements, synergies with stakeholders and challenges in anthrax disease management.

**Qualitative analysis**: qualitative data were transcribed in Microsoft^®^ Word (Ms Word) and analyzed for content along thematic areas. In transcribing the FGD, the narratives were read and reread and compared to audio files before transferring to Ms Word along the topic guides. For triangulation, we crosschecked narratives from key informants with those of FGDs to identify divergent or supporting views. Illustrative comments and quotations that clearly represented themes were quoted verbatim.

**Ethical considerations**: the protocol was reviewed by Kenya´s Ministry of Health and determined to be a response to an acute public health event. Thereafter, we sought and obtained authorization from Narok County public health authorities to engage the community in the investigation. Informed verbal consent was obtained from all respondents before data collection and discussions. Upon data entry, we de-identified participants on the questionnaires and transcripts and all information was kept confidential. All survey tools were kept under lock and key.

## Results

### KAP survey quantitative findings

**Socio-demographic characteristics**: among 334 respondents, 56% (187/334) were males; mean age was 40.7±13.6 years and majority 58.7% (196/334) were from Ilkerin ward. Of these 80.8% (270/334) had primary level of education. Participants had kept livestock for average duration of 20.1±13.3 years and almost all of the respondents 98.5% (329/334) kept more than one type of livestock.

**Figure 2:**
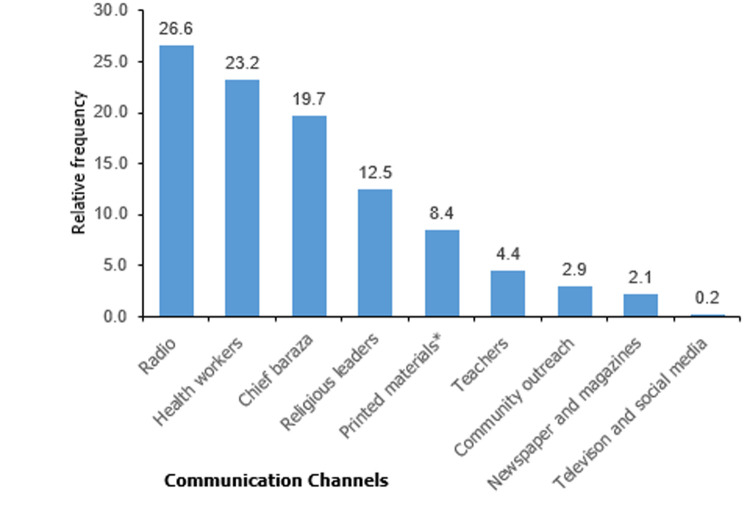
proportions of preferred communication channels for anthrax messages, Trans Mara East, 2018

**Knowledge analysis for anthrax**: almost all of the respondents 99% (331/334) had heard of disease anthrax. Participants´ knowledge of clinical signs in livestock were varied: 62.2% (206/331) mentioned bleeding from body orifices; 4.2% (14/331) reported sudden death in livestock while 21.5% (71/331) were unaware of any clinical signs. For humans, 95.8% (317/331) indicated skin lesions and 9.4% (31/331) gastroenteritis symptoms (Table 1). Furthermore, 43% (141/328) of respondents (missing values n=3) considered themselves not well informed of the disease and desired more information. The preferred communication channels were: radio 42% (139/331), professional workers 23.3% (121/331), community barazas and outreach 22.6% (118/331), religious leaders 19.6% (65/331) among others (Figure 2). Up to 91.8% (304/331) recognized anthrax as a zoonoses. Although 84% (278/331) responded to know the cause of anthrax, only 14% (39/278) identified germs (Table 1). The main transmission routes for infection were considered as eating meat of dead carcasses by 82.5% (273/331), contact with infected tissues by 64.4% (213/331) and inhalation of bacterial spores by 4.7% (8/331) of the participants (Table 1).

**Table 1:** knowledge of anthrax among respondents given as numbers (%), Trans Mara East, February 2018 (n = 331)

Characteristic	Frequency (n)	Proportion (%)
**Overall knowledge**		
Adequate	178	53.8
Inadequate	143	46.2
**Cause of anthrax n=278**		
Eating meat from dead-animal	273	82.5
Germs	39	14.0
Skinning dead animal	75	27.0
Grazing animal in burial sites	143	51.4
Bad air	32	11.5
Witchcraft	1	0.4
**Species affected by anthrax n=329**		
Human	329	100.0
Cow	304	91.8
Goat	136	41.1
Sheep	137	41.4
**Transmission mode in humans**		
Contact with infected tissues	213	64.4
Eating meat from dead carcasses	273	82.5
Inhalation of bacterial spores	8	2.4
**Clinical signs in human**		
Skin lesions	317	95.8
Severe cough	3	0.9
Severe diarrhea	31	9.4
Don´t know	6	1.8
**Preventive measures in humans**		
Eating inspected meat	179	54.1
Not skinning suspect animals	102	30.8
Avoid contact dead-animal fluid	117	35.4
Environmental hygiene	30	9.1
Burying dead animals	129	39.0
**Clinical signs in dead animals**		
Bleeding from orifices	206	62.2
Sudden death	14	4.2
Absent rigor mortis	25	7.6
Rapid decomposition	17	5.1
Bloating	96	29.0
Don´t know	71	21.5
**Preventive measures in animals**		
Knew livestock vaccination	304	91.8
Didn´t know	27	8.2

Most of the respondents, 91.8% (304/331), knew of livestock vaccination as preventive measure in animals and 94.9% (314/331) knew of control measures to protect humans from anthrax. These control measures included: eating inspected meat 54.1% (179/331), burying dead animals 39% (129/331) and avoiding contact with fluid from dead animals 35.4% (117/331) (Table 1). The median knowledge score computed from 34 item questions was 15. About half of the respondents, 53.8% (178/331) attained ≥ the median score and were classified as having adequate knowledge. Greater knowledge was recorded to increase with age, education level and duration of rearing livestock. Sex (χ^2^ = 17.78; p = 0.001) and Religion (χ^2^ = 3.48; p = 0.035) were also significant factors associated with knowledge (Table 2).

**Table 2:** total knowledge score of anthrax (n = 331) in Trans Mara East Narok, Kenya, 2018

Variable	Total, n (%)	Adequate knowledge, n (%)	Inadequate knowledge, n (%)	Median score 15/34	Chi square value	p-value	
**Age years**							
<25	28(8.5)	8(2.4)	20(6.1)	11			
25-44	190(57.4)	98(29.6)	92(27.8)	15	2.25	0.007	
45-64	88(26.6)	55(16.6)	33(10.0)	15.5			
65+	25(7.6)	17(5.2)	8(2.4)	15			
**Sex**							
Male	186(56.2)	119(36.0)	67(20.2)	15	17.78	**<0.001**	
Female	145(43.8)	59(17.8)	86(26.0)	14			
**Occupation**							
Casual	1(0.3)	1(0.3)	0(0)	15			
Farmer	305(92.2)	170(51.4)	135(40.8)	15	10.06	**0.018**	
Formal employed	3(0.9)	2(0.6)	1(0.3)	17			
Self employed	22(6.7)	5(1.5)	17(5.2)	12.5			
**Education level**							
No formal	43(13,0)	24(7.3)	19(5.7)	15			
Primary	267(80.7)	140(42.3)	127(38.4)	15	3.43	0.488	
Secondary	14(4.2)	8(2.4)	6(1.8)	18			
College	2(0.6)	2(0.6)	0(0)	18			
Adult education	5(1.5)	4(1.2)	1(0.3)	18			
**Religion**							
Christian	272(82.2)	153(46.2)	119(36.0)	15			
Muslim	2(0.6)	1(0.3)	1(0.3)	13	8.58	**0.035**	
None	51(15.4)	24(7.3)	27(8.1)	14			
Other	6(1.8)	0(0)	6(1.8)				
**Kept animals**							
Yes	329(99.4)	178(53.8)	151(45.6)	15	0.67	0.412	
No	2(0.6)	0(0)	2(0.6)	6.5			
**Livestock keeping years n=303**							
<10	66(21.8)	19(6.3)	47(15.5)	13			
10-29	155(51.2)	85(28.1)	70(21.1)	15	27.55	**<0.001**	
30+	82(27.1)	59(19.5	23(7.6)	16			

**Analysis of practices and attitudes with relevance to anthrax**: following sudden death, 61.1% (201/329) of respondents (missing values n = 2) reported that they would skin animals before burial while 28.8% (95/329) would either burn or bury. Only 4.6% (15/329) would contact animal health service provider (AHSP) for professional advice while 5.5% (18/329) would share the meat and/or give to dogs after removing the skin. Among the 59.5% (195/328) who slaughtered animals at home, only 11.8% (23/195) sought meat inspection services: 6.2% (12/195) from AHSP; 4.6% (9/195) from public health officers and 1.0% (2/195) sought community elders. Treatment of sick animals was co-shared between farmers themselves at 52.9% (172/325) and AHSP at 47.1% (153/325) in the investigation area (missing values n = 6). When animals showed poor signs for recovery, 85.5% (282/330) of the respondents would wait for its death and bury as others salvaged for hide and meat: 6.7% (22/330) would slaughter, sell or offer free meat; 1.5% (5/330) would slaughter and remove skin before burying and 6.4% (21/330) slaughter and bury. Only 59% (194/329) of the respondents (missing values n = 2) reported regular livestock vaccination against anthrax. More than 92% (307/331) considered anthrax a serious disease. Following anthrax outbreak, 96.4% (319/331) would call AHSP as well as (319/331) seeking care at a health facility and less than 3% (9/331) would resort to herbs while 0.3% (1/331) would buy drugs from chemists. The median score from 16 item questions regarding practices related to anthrax was 11 and a total of 142/331 (42.9%) scored ≥ median, thus categorized as having good practice. Surprisingly, respondents with secondary and college education (χ^2^ = 10.68; p = 0.031) did not translate to good practices. Significant differences in practices were also reported among age groups (χ^2^ = 11.68; p = 0.009), sex (χ^2^ = 7.46; p = 0.006) and duration of rearing livestock (χ^2^, 8.55; p = 0.013) (Table 3).

**Table 3:** total practice score of anthrax (n = 331) in Trans Mara East Narok, Kenya, 2018

Variable	Total, n (%)	Good practice, n (%)	Poor practice, n (%)	Median score 11/16	Chi square value	p-value
**Age (years)**						
<25	28(8.5)	23(6.9)	5(1.5)	11		
25-44	190(57.4)	104(31.4)	86(26.0)	11	11.68	**0.009**
45-64	88(26.6)	44(13.3)	44(13.3)	10		
65+	25(7.6)	18(5.4)	7(2.2)	11		
**Sex**						
Male	186(56.2)	94(28.4)	62(27.8)	11	7.46	**0.006**
Female	145(43.8)	95(28.7)	50(15.1)	11		
**Occupation**						
Casual	1(0.3)	1(0.3)	0(0)	12		
Farmer	305(92.2)	171(51.7)	134(40.5)	11	2.10	0.552
Formal employed	3(0.9)	2(0.6)	1(0.3)	11		
Self employed	22(6.7	15(4.5)	7(2,2)	11.5		
**Education level**						
No formal	43(13,0)	30(9.1)	13(3.9)	11		
Primary	267(80.7)	152(45.9)	115(34.7)	11	10.68	0.031
Secondary	14(4.2)	6(1.8)	8(2.4)	10		
College	2(0.6)	1(0.3)	1(0.3)	10.5		
Adult education	5(1.5)	0(0)	5(1.5)	10		
**Religion**						
Christian	272(82.2)	155(16.8)	117(35.3)	11		
Muslim	2(0.6)	2(0.6)	0(0)	11.5	1.63	0.653
None	51(15.4)	29(0.8)	22(6.6)	11.0		
Other	6(1.8)	3(0.9)	3(0.9)	10.5		
**Kept animals**						
Yes	329(99.4)	188(56.8)	141(42.6)	11	0.04	0.839
No	2(0.6)	1(0.3)	1(0.3)	10.5		
**Livestock keeping years n=303**						
<10	66(21.8)	47(15.5)	19(6.3)	11		
10-29	155(51.2)	86(28.4)	69(22.8)	11	8.55	**0.013**
30+	82(27.1)	39(12.9)	43(14.2)	10		

**Qualitative findings from key informant interviews and focus group discussions**: we held three FGDs in three villages and six key informant interviews. Qualitative assessments focussed on emerging themes on knowledge, attitudes and practices as well as challenges in addressing anthrax outbreaks. The disease had a local name from FGDs; *“burasta”* which translates to curse of sudden death without warning signs. A clinician in a local health facility considered the area a “hotspot” for anthrax. *“(Anthrax) burasta is like a curse come here after rains pastures and animals are in good body condition ...only to wake up and find them dead*” (a male participant in FGD1).

*“Anthrax is endemic here the recent outbreak (January 2018) has been mild have had no in-patients and no fatalities, in 2014 we had all three forms of anthrax even fatalities I don´t have the records at hand but I can recall we made several referrals to Kilgoris sub-county hospital .*.” (clinical officer at local health centre from qualitative interviews the participants summed the cycle of infection thus);

*“These days´ people don´t plant ‘mocheket´ (a cactus like plant) anymore still graze in areas where animals died exposes our animals to same germs”* (Female participant in FGD2).

In all group discussions, participants were unaware of anthrax transmission from infected hides and skins. *“Almost every household has a skin spare one for a visitor Sleeping on skin is not sign of poverty makes your cattle to multiply shows love for them* (Male participant in FGD 2).

*“My two sons sleep on the hides had skin lesions on face told it is burasta” we did not eat meat nor touch it. I don´t know how they become sick”* (Female participant in FGD1).

Dead and dying animals were unlikely to be disposed off as the community salvaged for meat, hides and skins. The community elaborated traditional procedures for ascertaining meat safety; *We test meat from dead animals using ants. I cut and throw a piece of liver to black ants (sumonyot). If they eat....meat is safe. If they walk away it is bad. Another test I do is using Basiriat leaves. If placed on shoulder meat, if the meat is bad it will wilt away after touching blood* (Male participant in FGD 1).

*“Our husbands come home with meat and force us to cook, we cannot refuse to obey their orders. They will eat with children. I boil the meat and throw away the first water after boiling, then I boil again”* (Female participant in FGD2).

Though the community was aware of livestock vaccination as preventive measure, government funded vaccinations in the area were rare. Recent livestock vaccinations were largely privately provided. The discussants cited a lapse of five years since the last government-funded vaccinations. A number of reasons were cited for failure to vaccinate animals including being: unaware of need for anthrax vaccinations; considered vaccination expensive and lack of publicity for the exercise or vaccination site being far from their homes.

*“We have never held any animal vaccination leave alone anthrax vaccination since advent of county government in 2013 despite diseases Foot and mouth disease, black quarter, contagious pleuropneumonia occasionally striking our herds, we are a forgotten lot”* (Male participant in FGD 3).

During the recent outbreak, the health teams´ response centered on patient care including antibiotic prophylaxis, decontamination, community health talks and enhanced surveillance. The communities summed the seriousness of burasta”.

*“If there is an outbreak of anthrax, we walk to the nearest administrative officer, village elder or chief, who relays messages to appropriate government authorities, “burasta” means death* (Male participant in FGD 2).

The veterinary team enumerated barriers to anthrax outbreak response comprising of staffing, cold chain management and transport logistics. He expressed frustrations urging for prompt reports from the community and recommending livestock vaccinations while addressing community health talks when he did not have resources to respond. He lamented:

*“I am expected to travel to Narok town (county headquarters) to lobby for supplies. I have no transport and the public means are rare and even if I was provided with vaccines, electricity supply has been disconnected..... Local community blames me for inaction! Only two of us cover the whole sub-county”* (Local veterinary officer for collaboration strategies, the community health talks were conducted both in health centers and villages. The area administration chief noted).

*“I want to say that with intensive community education and village barazas (public community meetings) we have been able to get more messages into community members to stop feasting on dead and dying animals”* (Local administration official).

## Discussion

The investigation demonstrates focal areas for anthrax prevention and control. The results do suggest that societal human behaviour played a key role in transmission of anthrax outbreaks [7]. Majority of respondents did not identify germs to cause anthrax leading to improper methods of carcass handling and disposal. The customary opening of dead animals to salvage for meat, skin and hides causes vegetative bacilli to sporulate when exposed to air which consequently contaminates the soil and water before being spread further by scavenger birds and animals resulting in a cycle of infection [8]. Predominantly, all households kept livestock. The persistence of these practices in some communities where animals are a great asset and the sudden death of animals with no prospect of compensation necessitates salvage of anthrax-infected animals as communal empathy for the loss! Similar findings have been reported in Ghana and Zimbabwe [7,9]. This human behaviour has occasioned a correlation between human and animal disease [1,10]. The lack of formal employment may limit access to regular income and therefore capacity to access meat protein from markets necessitating the consumption of dead and or dying animals as reported in a case control study of cutaneous anthrax in Zimbabwe [11]. The recognition of major clinical signs and transmission pathways in human and livestock does suggest public health education could be potentially effective in preventing anthrax. Respondents who knew anthrax to be zoonotic (91%) was higher than reported in Ghana and Zambia at 64.2% [4,7]. These differences could be attributed to fact that the investigation was conducted during an outbreak which minimized recall bias.

The inadequate knowledge on anthrax by half of the population could be attributed to the low levels of education in which less than 5% of the respondents had secondary and college education. This compares with a similar cross-sectional study in Zambia [4]. Education imparts one with capacity to access information and comprehend health messages. The low levels of education likely meant majority of respondents had poor access to media information and poor comprehension and compliance to health education messages [12]. However, the poor practices among respondents with secondary and college level of education is surprising. It does suggest other socio-cultural norms may need further evaluation. The preferred use of radio and community outreaches needs to focus on aforementioned hazards and evaluated as effective channels for communication. Other studies have reported the same channels as sources of information to the community on zoonotic diseases [12,13]. Religion (χ^2^ = 8.58; p-value = 0.035) does seem to significantly be able to contribute to understanding of the disease and therefore religious forums could be used to spread public health messages to the community. The community treatment of sick animals could be related to inadequacy of veterinary services. Veterinary officers enumerated barriers hindering effective anthrax outbreak response and prevention. Similar findings have been described in studies for re-emergence of anthrax and other zoonoses [1,12]. The communities result to ethno-veterinary knowledge such as use of observation, herbs, and ants to define meat safety and planting of poisonous plants in animal burial sites. Similar findings have been made in the case of rift valley fever disease among the communities in Baringo and Ijara regions of Kenya [12]. Controlling infection of anthrax in animals is key to control of human anthrax [1]. Good veterinary practice, burying of animal carcasses, use of effective decontamination and disinfection procedures as well as immunization of animals and education of animal owners will improve outcomes in the area. Knowledge of animal vaccination as a preventive measure needs to be translated into actionable regular vaccination. The challenges and barriers may have been exacerbated by changes in veterinary governance in Kenya following devolution and decentralization of veterinary services. While the survey did not purport to evaluate veterinary system, the potential impact on anthrax and other livestock diseases in the context of evolving veterinary governance needs considered evaluation.

**Limitations**: the assessment of current knowledge and practices may have been influenced by the ongoing health promotion messages to control the outbreak. However, this was useful in overcoming recall bias. Our findings may contain information errors related to desirability bias in which knowledgeable respondents may have stated what was desirable rather than what they engaged in. Adherence to good practice in livestock production and anthrax control was self-reported by the respondents rather than observed by the investigators. It should be borne these findings are specific to Trans Mara East and may not be generalizable to other areas with different social conditions. Despite the limitations, by triangulation of quantitative and qualitative findings, we strived to ensure internal validity and reliability. Our investigation therefore highlights elements that would expose the community to anthrax in the event of an outbreak.

## Conclusion

Though more than half of respondents were found to have adequate knowledge, this did not translate to good practice. Undesirable practices related to skinning dead-animal and treatment of sick animals increased their risk of exposure to anthrax. This could be due to demographic differences such as sex, education level and age which may impact on any prevention and control measures. Enhanced public health education and targeted interventions with one health approach by relevant government bodies is highly recommended for effective prevention and control of anthrax. Emphasizes should be on hazards associated with ubiquitous salvages from dying and or dead-animal. Animal owners should ensure sick and or dying animals are not skinned, slaughtered or butchered for meat consumption. Veterinary services were inadequate and investments in terms of staffing, logistical support is vital in ensuring rapid detection and control of outbreaks in livestock populations. It is imperative the county government of Narok improves anthrax control activities in the area. The qualitative findings are likely to help public health authorities design strategies that target some of the practices, are culture specific and appropriate to community behaviour and ultimately contribute to improved prevention and control measures.

### What is known about this topic

- There is correlation between animal and human anthrax;
- Risks emanate from consumption of meat from suspected animal anthrax cases;
- Public health education is an important factor in controlling anthrax outbreaks.

### What this study adds

- Knowledge of anthrax among the Trans Mara residents;
- Undesirable cultural practices that aid in transmission of anthrax;
- Communication channels for public health education in the community.
